# Risk of Nephrotic Syndrome following Enteroviral Infection in Children: A Nationwide Retrospective Cohort Study

**DOI:** 10.1371/journal.pone.0161004

**Published:** 2016-08-10

**Authors:** Jiun-Nong Lin, Cheng-Li Lin, Chi-Hui Yang, Ming-Chia Lin, Chung-Hsu Lai, Hsi-Hsun Lin, Chia-Hung Kao

**Affiliations:** 1 Department of Critical Care Medicine, E-Da Hospital, I-Shou University, Kaohsiung, Taiwan; 2 School of Medicine, College of Medicine, I-Shou University, Kaohsiung, Taiwan; 3 Division of Infectious Diseases, Department of Internal Medicine, E-Da Hospital, I-Shou University, Kaohsiung, Taiwan; 4 Management Office for Health Data, China Medical University Hospital, Taichung, Taiwan; 5 College of Medicine, China Medical University, Taichung, Taiwan; 6 General Education Center, Meiho University, Pingtung, Taiwan; 7 Department of Nuclear Medicine, E-Da Hospital, I-Shou University, Kaohsiung, Taiwan; 8 Graduate Institute of Clinical Medical Science and School of Medicine, College of Medicine, China Medical University, Taichung, Taiwan; 9 Department of Nuclear Medicine and PET Center, China Medical University Hospital, Taichung, Taiwan; 10 Department of Bioinformatics and Medical Engineering, Asia University, Taichung, Taiwan; University of Hong Kong, HONG KONG

## Abstract

**Purpose:**

Nephrotic syndrome is a common chronic illness encountered during childhood. Infections have been identified as a cause of nephrotic syndrome. The aim of this study was to evaluate the association between enteroviral infection and nephrotic syndrome.

**Methods:**

A nationwide retrospective cohort study was conducted by analyzing data from the National Health Insurance Research Database in Taiwan. Children aged <18 years with enteroviral infection were enrolled. Non-enterovirus-infected children were randomly selected as the comparison cohort. The primary endpoint was the occurrence of nephrotic syndrome.

**Methods:**

This study included 280,087 enterovirus-infected children and 280,085 non-enterovirus-infected children. The mean age of the enterovirus-infected children was 2.38 years, and 53.7% of these children were boys. The overall incidence densities of nephrotic syndrome for enterovirus- and non-enterovirus-infected children were 2.65 and 2.21 per 10,000 person-years, respectively. The enterovirus-infected cohort had a higher cumulative incidence of nephrotic syndrome than did the non-enterovirus-infected cohort (log-rank test, p = 0.01). Multivariable analyses revealed that children with enteroviral infection were significantly associated with an increased risk of nephrotic syndrome compared with those without enteroviral infection (adjusted hazard ratio, 1.20; 95% confidence interval, 1.04–1.39; p = 0.01), particularly in children infected with coxsackievirus. Subgroup analyses revealed that enterovirus-infected girls, children of blue-collar workers, and children without allergies had a higher risk of nephrotic syndrome than did children in the non-enterovirus-infected cohort.

**Conclusion:**

This study revealed a significant association between enteroviral infection and nephrotic syndrome. Additional studies elucidating the role and pathogenesis of enterovirus in nephrotic syndrome are warranted.

## Introduction

Nephrotic syndrome, characterized by proteinuria, hypoalbuminemia, and generalized edema, is among the most common chronic kidney diseases encountered during childhood [1,2]. It can be idiopathic or provoked by systemic diseases (eg, diabetes mellitus, systemic lupus erythematosus, amyloidosis, and cancers), drugs, and infections [2]. The incidence and prevalence of idiopathic nephrotic syndrome are 1–7 and 16 per 100,000 children, respectively [2,3]. The long-term prognosis of nephrotic syndrome is generally favorable; however, its characteristics and related therapy can massively influence childhood development and quality of life in children. Refractory nephrotic syndrome in some patients may progress to end-stage renal disease [2,4].

Enteroviruses include diverse RNA viruses classified in the *Picornaviridae* family. More than 90 distinct viral serotypes, including enteroviruses, polioviruses, coxsackieviruses, rhinoviruses, and echoviruses, have been identified [5,6]. Enteroviral infections are common among children worldwide. The Centers for Disease Control and Prevention estimated that approximately 10–15 million infections of non-polio enteroviruses occurred in the United States annually [7]. Enteroviral infections present a wide range of manifestations, such as hand-foot-and-mouth disease, meningitis, encephalitis, conjunctivitis, herpangina, myocarditis, pericarditis, acute flaccid paralysis, and inflammatory muscle disease [5–7].

Several pathogens, including hepatitis B virus, hepatitis C virus, human immunodeficiency virus (HIV), malaria, syphilis, and toxoplasmosis have been known to be associated with nephrotic syndrome [2]. Over the last 50 years, enteroviruses, particularly coxsackieviruses, have been sporadically reported to be causative pathogens of glomerulonephritis and nephrotic syndrome [8–14]. Receptors for coxsackievirus have been identified in the human kidney [15,16]. However, studies on the association between enteroviral infection and renal diseases are limited. According to our review of relevant literature, no epidemiologic study has investigated the incidence of nephrotic syndrome following enteroviral infection. Therefore, we conducted a nationwide retrospective cohort study to determine the subsequent risk of nephrotic syndrome in children infected with enteroviruses by analyzing data from the National Health Insurance Research Database (NHIRD) of Taiwan.

## Materials and Methods

### Data sources

The National Health Insurance (NHI) program of Taiwan is a single-payer and obligatory health care program initiated in 1995, covering more than 99% of the 23.75 million residents of Taiwan [17]. The NHIRD contains research data released from the NHI program. In this retrospective cohort study, we used research data from the NHI reimbursement claims. To comply with data privacy regulations, personal identities were encrypted and all NHIRD data were analyzed in a deidentified manner. We used a dataset from the NHIRD that consists of a random selection of 50% of all insured children aged <18 years during 2000–2008 in Taiwan. Diagnostic codes of the International Classification of Diseases, Ninth Revision, Clinical Modification (ICD-9-CM) were used to identify the diseases.

### Ethics statement

The NHIRD encrypts patient personal information to protect privacy and provides researchers with anonymous identification numbers associated with relevant claims information, including sex, date of birth, medical services received, and prescriptions. Therefore, patient consent is not required to access the NHIRD. This study was approved to fulfill the condition for exemption by the Institutional Review Board (IRB) of China Medical University (CMUH104-REC2-115). The IRB also specifically waived the consent requirement.

### Study design

The enterovirus-infected cohort included patients aged <18 years with enteroviral infection diagnosed during 2000–2007. The enteroviral infections included enteritis due to enterovirus (ICD-9-CM code 008.67), meningitis due to enterovirus (ICD-9-CM code 047), other enterovirus diseases of central nervous system (ICD-9-CM code 048), specific diseases due to coxsackievirus (ICD-9-CM code 074), echovirus infection in conditions classified elsewhere and of unspecified site (ICD-9-CM code 079.1), and coxsackievirus infection in conditions classified elsewhere and of unspecified site (ICD-9-CM code 079.2). Children with poliovirus infection (ICD-9-CM code 045) and rhinovirus infection (ICD-9-CM code 079.3) were not included in this study. The date of enteroviral infection was defined as the index date. For each child with enteroviral infection, one random child without enteroviral infection matched by sex, age (within a 1-year interval), urbanization of residential area, parental occupation, and the year of enteroviral infection was enrolled to form the comparison cohort. Children whose date of birth or sex were missing in the records and those with pre-existing nephrotic syndrome (ICD-9-CM code 581) were excluded from this study. In addition, we excluded patients who had diseases that were possibly associated with secondary nephrotic syndrome, namely HIV infection (ICD-9-CM codes 042–044), all diabetes mellitus (ICD-9-CM code 250), autoimmune diseases except type I diabetes mellitus (ICD-9-CM codes 710.0, 710.1, 710.2, 710.3, 714, 725), hepatitis B virus infection (ICD-9-CM codes 070.2–070.3), hepatitis C virus infection (ICD-9-CM codes 070.41, 070.44, 070.51, 070.54, 070.7, V02.62), cancer (ICD-9-CM codes 140–208), amyloidosis (ICD-9-CM codes 277.3), sarcoidosis (ICD-9-CM codes 135, 321.4), herpes zoster infection (ICD-9-CM code 053), and syphilis (ICD-9-CM codes 090–097, 104.0, 647.0).

### Primary outcome

The study outcome was a diagnosis of nephrotic syndrome during an 8-year follow-up period. Each child was followed from the index date until either the development of nephrotic syndrome, withdrawal of the insurance program, death, or December 31, 2008, whichever occurred earlier. Comorbidities determined for each child included atopic dermatitis (ICD-9-CM code 691.8), allergic rhinitis (ICD-9-CM code 477), and bronchial asthma (ICD-9-CM code 493).

### Statistical analyses

Sociodemographic variables analyzed in this study included age, sex, urbanization level, and parental occupation (white-collar jobs, blue-collar jobs, and others). The urbanization level was categorized into 4 levels that were based on the population density of the residential area [18]. Level 1 was the most urbanized and level 4 was the least urbanized. Distributions of sociodemographic variables in the enterovirus-infected and non-enterovirus-infected cohorts were examined using chi-square tests for categorical variables and Student *t* tests for continuous variables. Kaplan-Meier analyses were performed for determining the cumulative incidence of nephrotic syndrome, and the log-rank test was used to calculate the difference between the enterovirus- and non-enterovirus-infected cohorts. The incidence density of nephrotic syndrome was estimated by dividing the number of nephrotic syndrome with the number of person-years for different risk factors stratified by age, sex, urbanization level, parental occupation, and comorbidities. Univariable and multivariable Cox proportion hazard regression models were used to examine the effect of enteroviral-infection on the risk of nephrotic syndrome and reported as a hazard ratio (HR) with a 95% confidence interval (CI). Multivariable models were used to determine the risk of nephrotic syndrome after adjustment for the variables. SAS statistical software package (Version 9.3 for Windows; SAS Institute, Inc., Cary, NC, USA) was used for analyzing the data. A p-value less than 0.05 was considered statistically significant.

## Results

This study included 280,087 enterovirus-infected children and 280,085 non-enterovirus-infected children. Table 1 presents the sociodemographic variables and comorbidities of both cohorts. The mean age of the enterovirus-infected children was 2.38 years (standard deviation, 1.87 years). Boys accounted for 53.7% of all patients and 55% of children were <2 years. More than half of the children (55.5%) lived in higher urbanization regions (level 1 and level 2), and parents of 64% of the children were white-collar workers. Compared with the non-enterovirus-infected cohort, all allergies were more prevalent in the enterovirus-infected cohort (p <0.001).

**Table 1:** Sociodemographics and comorbidity in children with and without enterovirus infection.

	Non-enteroviral infection (n = 280,085)	Enteroviral infection (n = 280,087)	p value
**Age, mean ± SD (years)**	2.38 ± 1.92	2.38 ± 1.87	0.48
**Stratified age**			0.99
< 2	154,003 (55.0%)	154,005 (55.0%)	
≥2	126,082 (45.0%)	126,082 (45.0%)	
**Sex**			0.99
Girl	129,825 (46.4%)	129,826 (46.4%)	
Boy	150,260 (53.7%)	150,261 (53.7%)	
**Urbanization**^†^			0.99
1 (highest)	72,192 (25.8%)	72,192 (25.8%)	
2	83,116 (29.7%)	83,116 (29.7%)	
3	54,582 (19.5%)	54,582 (19.5%)	
4 (lowest)	70,195 (25.1%)	70,197 (25.1%)	
**Parental occupation**			0.99
White collar	179,215 (64.0%)	179,217 (64.0%)	
Blue collar	67,818 (24.2%)	67,818 (24.2%)	
Others^‡^	33,052 (11.8%)	33,052 (11.8%)	
**Comorbidity**			
Atopic dermatitis	8135 (2.90%)	11,941 (4.26%)	<0.001
Allergic rhinitis	24,722 (8.83%)	41,432 (14.8%)	<0.001
Bronchial asthma	16,157 (5.77%)	28,495 (10.2%)	<0.001

SD, standard deviation.

^†^ The urbanization level was categorized by the population density of the residential area into 4 levels; level 1 was the most urbanized and level 4 was the least urbanized.

^‡^ Other occupations included primarily retired, unemployed, or low income populations.

**Fig 1:**
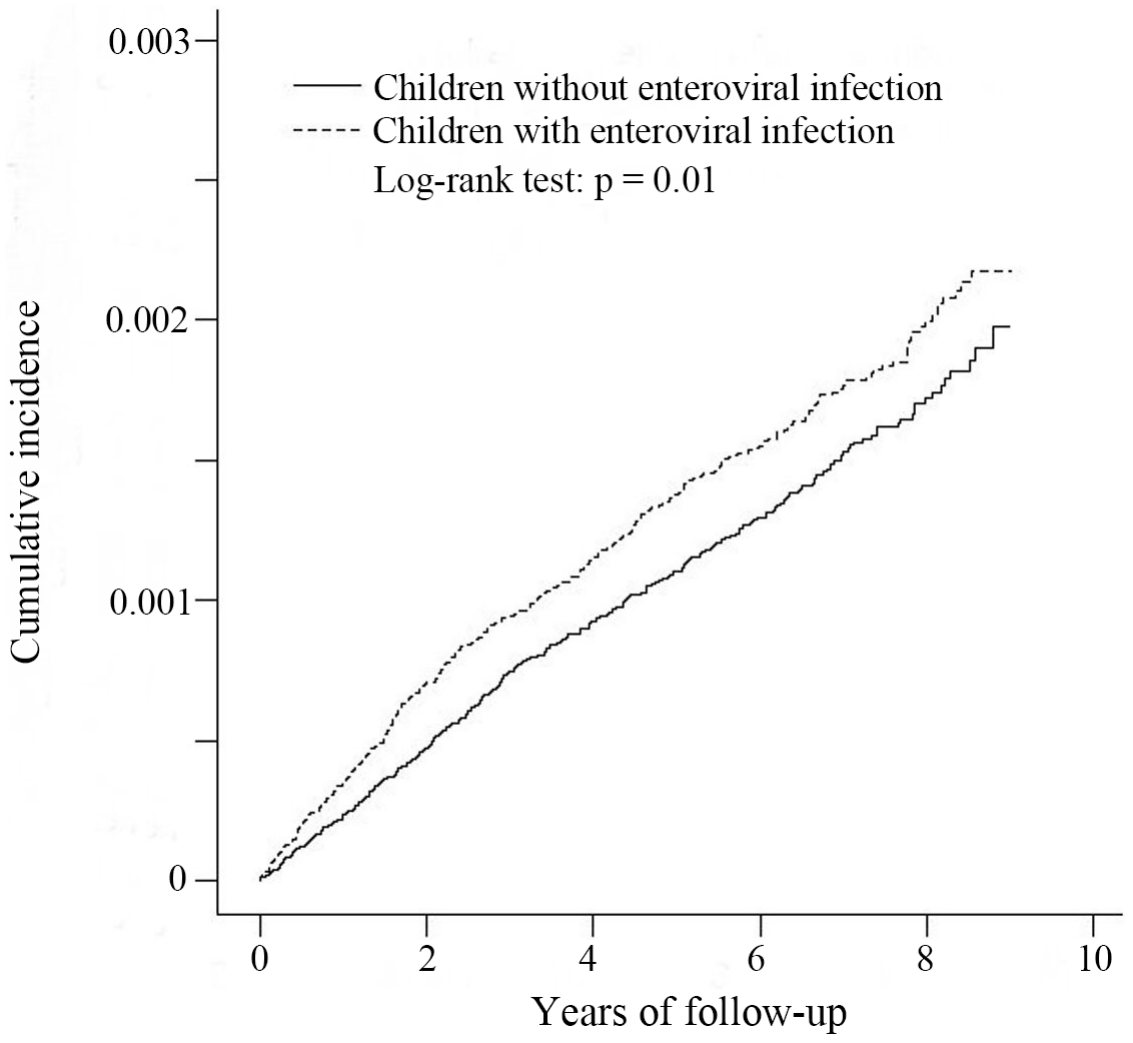
The Kaplan-Meier analysis of cumulative incidence of nephrotic syndrome for patients with (dashed line) or without (solid line) enteroviral infection.

The mean (± standard deviation) duration of follow-up was 5.53 (± 2.26) years and 5.51 (± 2.26) years in the enterovirus- and non-enterovirus-infected cohorts, respectively. Kaplan-Meier analyses revealed that the enterovirus-infected cohort had a higher cumulative incidence of nephrotic syndrome than did the non-enterovirus-infected cohort (log-rank test, p = 0.01, Fig 1). The overall incidence of nephrotic syndrome for enterovirus-infected and non-enterovirus-infected children were 2.65 and 2.21 per 10,000 person-years, respectively (Table 2). After adjustment for age, sex, urbanization level, parental occupation, and allergic diseases, the multivariable analyses revealed that children with enteroviral infection were associated with an increased risk of nephrotic syndrome compared with those without enteroviral infection (adjusted HR, 1.20; 95% CI, 1.04–1.39; p = 0.01). Boys were at a higher risk for nephrotic syndrome development than were girls (adjusted HR, 1.23; 95% CI, 1.06–1.42). Compared with those living in the most urbanized levels, children in the third highest urbanized level had a higher risk for the nephrotic syndrome development (adjusted HR, 1.31; 95% CI, 1.06–1.62). However, the age at the diagnosis of enteroviral infection, parental occupation, and allergic disease demonstrated no significant difference in the incidence of nephrotic syndrome.

**Table 2:** The incidence and hazard ratio and 95% confidence intervals for nephrotic syndrome.

Variable	Event	Person-years	IR	Crude HR (95% CI)	p value	Adjusted HR (95% CI) ^†^	p value
**Enteroviral infection**							
No	341	1,542,399	2.21	1 (Reference)		1 (Reference)	
Yes	410	1,548,686	2.65	1.20 (1.04–1.38)*	0.01	1.20 (1.04–1.39)*	0.01
**Age, year**							
<2	411	1,705,568	2.41	1 (Reference)		1 (Reference)	
≥2	340	1,385,517	2.45	1.02 (0.88–1.17)	0.83	1.03 (0.89–1.19)	0.73
**Sex**							
Girl	310	143,157	2.17	1 (Reference)		1 (Reference)	
Boy	441	1,659,729	2.66	1.23 (1.06–1.42)**	0.006	1.23 (1.06–1.42)**	0.006
**Urbanization**^‡^							
1 (highest)	168	791,385	2.12	1 (Reference)		1 (Reference)	
2	225	917,750	2.45	1.16 (0.95–1.41)	0.16	1.18 (0.96–1.44)	0.12
3	166	605,768	2.74	1.29 (1.04–1.60)*	0.02	1.31 (1.06–1.62)*	0.01
4 (lowest)	192	776,183	2.47	1.17 (0.95–1.44)	0.14	1.21 (0.97–1.49)	0.09
**Parental occupation**							
White collar	489	1,971,554	2.48	1 (Reference)		1 (Reference)	
Blue collar	183	768,159	2.38	0.96 (0.81–1.14)	0.67	0.95 (0.79–1.13)	0.56
Others^‡^	79	351,373	2.25	0.91 (0.71–1.15)	0.41	0.88 (0.70–1.12)	0.31
**Atopic dermatitis**							
No	724	3,000,304	2.41	1 (Reference)		1 (Reference)	
Yes	27	90781	2.97	1.20 (0.82–1.76)	0.36	1.16 (0.92–1.46)	0.40
**Allergic rhinitis**							
No	657	2,745,871	2.39	1 (Reference)		1 (Reference)	
Yes	94	345,214	2.72	1.13 (0.91–1.40)	0.27	1.18 (0.80–1.74)	0.22
**Asthma**							
No	699	2,848,313	2.45	1 (Reference)		1 (Reference)	
Yes	52	242,773	2.14	0.87 (0.66–1.15)	0.34	0.77 (0.57–1.04)	0.09

IR, incidence rate per 10,000 person-years; HR, hazard ratio; CI, confidence interval.

^†^ Adjusted for age, sex, urbanization level, parental occupation and comorbidity of atopic dermatitis, allergic rhinitis, and bronchial asthma.

^‡^ The urbanization level was categorized by the population density of the residential area into 4 levels; level 1 was the most urbanized and level 4 was the least urbanized.

* p <0.05,

** p <0.01.

The incidence rates of nephrotic syndrome for enterovirus- and non-enterovirus-infected cohorts were stratified by sex, age, urbanization level, parental occupation, and allergic disease (Table 3). Sex-specific risk analyses revealed that girls with enteroviral infection exhibited a significantly higher risk of nephrotic syndrome than did those who were not infected (adjusted HR, 1.36; 95% CI, 1.09–1.71). Enterovirus-infected children of blue-collar workers had a higher incidence of nephrotic syndrome than did those not infected (adjusted HR, 1.35, 95% CI, 1.01–1.81). The comorbidity-specific analyses revealed that children in the enterovirus-infected cohort who had no atopic dermatitis (adjusted HR, 1.18; 95% CI, 1.02–1.37), allergic rhinitis (adjusted HR, 1.25; 95% CI, 1.07–1.46), and bronchial asthma (adjusted HR, 1.22; 95% CI, 1.05–1.41) had a significantly higher risk of nephrotic syndrome than did children in the non-enterovirus-infected cohort.

**Table 3:** The risk of nephrotic syndrome associated with and without enteroviral infections in Cox proportional hazard regression.

	Non-enteroviral infection			Enteroviral infection			Adjusted HR^†^	
	Event	Person-years	IR	Event	Person-years	IR	(95% CI)	p value
**Age, years**								
<2	188	850,309	2.21	223	855,259	2.61	1.20 (0.99–1.45)	0.07
≥2	153	692,090	2.21	187	693,427	2.70	1.21 (0.97–1.50)	0.09
**Sex**								
Girl	131	714,347	1.83	179	717,010	2.50	1.36 (1.09–1.71)**	0.007
Boy	210	828,053	2.54	231	831,676	2.78	1.10 (0.91–1.33)	0.33
**Urbanization**^‡^								
1 (highest)	83	394,635	2.10	85	396,750	2.14	1.04 (0.77–1.41)	0.81
2	102	458,014	2.23	123	459,736	2.68	1.21 (0.93–1.58)	0.15
3	72	302,205	2.38	94	303,563	3.10	1.28 (0.94–1.74)	0.12
4 (lowest)	84	387,546	2.17	108	388,638	2.78	1.28 (0.96–1.70)	0.09
**Parental occupation**								
White collar	233	984,093	2.37	256	987,461	2.59	1.10 (0.92–1.32)	0.30
Blue collar	78	383,774	2.03	105	384,385	2.73	1.35 (1.01–1.81)*	0.04
Others	30	174,533	1.72	49	176,840	2.77	1.58 (1.00–2.50)	0.05
**Atopic dermatitis**								
No	333	1,506,190	2.21	391	1,494,115	2.62	1.18 (1.02–1.37)*	0.02
Yes	8	36,210	2.21	19	54,571	3.48	1.72 (0.75–3.93)	0.20
**Allergic rhinitis**								
No	303	1,414,880	2.14	354	1,330,991	2.66	1.25 (1.07–1.46)**	0.005
Yes	38	1,27,5120	2.98	56	217,695	2.57	0.87 (0.58–1.32)	0.52
**Bronchial asthma**								
No	322	1,455,645	2.21	377	1,392,668	2.71	1.22 (1.05–1.41)**	0.009
Yes	19	86754	2.19	33	156,018	2.12	0.96 (0.55–1.69)	0.89

IR, incidence rate per 10,000 person-years; HR, hazard ratio; CI, confidence interval.

^†^ Adjusted for age, sex, urbanization level, parental occupation and comorbidity of atopic dermatitis, allergic rhinitis, and bronchial asthma.

^‡^ The urbanization level was categorized by the population density of the residential area into 4 levels; level 1 was the most urbanized and level 4 was the least urbanized.

* p <0.05,

** p <0.01.

We further evaluated the risk of nephrotic syndrome stratified by the subtypes of enteroviral infections (Table 4). A significantly higher risk of nephrotic syndrome was observed in children infected with coxsackievirus (adjusted HR, 1.20; 95% CI, 1.04–1.39) than in non-enterovirus-infected children. Further analyses revealed that children with herpangina exhibited an increased risk of nephrotic syndrome (adjusted HR, 1.20; 95% CI, 1.03–1.40) whereas those with hand-foot-and-mouth disease did not (adjusted HR, 1.24; 95% CI, 0.97–1.57).

**Table 4:** Incidence and hazard ratio of nephrotic syndrome stratified by types of enteroviral infection.

Variable (ICD-9-CM)	No·	Event	Person-years	IR	Adjusted HR^†^ (95% CI)	p value
**Non-enteroviral infection cohort**	280,085	341	1,542,400	2.21	1 (Reference)	
**Subtype of enteroviral infection**						
Enteritis due to enterovirus (008.67)	29	0	100	0.00	-	
Meningitis due to enterovirus (047)	655	1	3773	2.65	1.19 (0.17–8.46)	0.86
Other enterovirus diseases of central nervous system (048)	180	0	1212	0.00	-	
Specific diseases due to coxsackievirus (074)	278,911	409	1,541,793	2.65	1.20 (1.04–1.39)*	0.01
Herpangina (074.0)	224,028	322	1,213,000	2.65	1.20 (1.03–1.40)*	0.02
Epidemic pleurodynia (074.1)	30	0	174	0.00	-	
Coxsackievirus carditis (074.2)	31	0	195	0.00	-	
Hand-foot-and-mouth disease (074.3)	51,606	83	305,970	2.71	1.24 (0.97–1.57)	0.09
Other specified diseases due to coxsackievirus (074.8)	3216	4	22,454	1.78	0.83 (0.31–2.22)	0.71
Echovirus infection in conditions classified elsewhere and of unspecified site (079.1)	77	0	352	0.00	-	
Coxsackievirus infection in conditions classified elsewhere and of unspecified site (079.2)	235	0	1456	0.00	-	

IR, incidence rate per 10,000 person-years; HR, hazard ratio; CI, confidence interval.

^†^ Adjusted for age, sex, urbanization level, parental occupation and comorbidity of atopic dermatitis, allergic rhinitis, and bronchial asthma.

* p <0.05.

## Discussion

This large retrospective cohort study revealed a 1.20-fold increased risk of nephrotic syndrome in children with enteroviral infections, particularly in those infected with coxsackievirus.

Enteroviral infections are very common in children. These viruses are associated with a broad spectrum of manifestations, ranging from minor febrile illness to severe and life-threatening diseases. For example, coxsackievirus infections in infants can result in hand-foot-and-mouth disease, myocarditis, meningitis, and encephalitis [19]. Coxsackievirus A24 and enterovirus 70 are associated with acute hemorrhagic conjunctivitis [20]. Recently, some high virulent strains of enteroviruses have emerged and became a severe threat to children worldwide. Enterovirus 71 causes large outbreaks of diseases in Asia, including hand-foot-and-mouth disease, myocaridits, pulmonary edema, acute flaccid paralysis, meningitis, and encephalitis (particularly brain stem encephalitis) [6,21]. A large outbreak of severe respiratory illness caused by enterovirus 68 occurred in the United States and Canada during 2014 [22]. The Centers for Disease Control and Prevention reported 1,153 patients with severe respiratory illness infected by enterovirus 68, including 14 deaths in children [23].

The involvement of kidney in patients infected with enterovirus had been reported since the 1960s. Burch and Colcolough [8] detected coxsackievirus B antigen in the kidney and heart of a patient with nephritis and pancarditis, and several reports of similar findings have since been described [9–11]. Coxsackieviruses B were reported to infect human glomerular and tubular cell lines, produce cytopathologic changes, and impair the phagocytic and contractile activity of mesangial cells [24]. The coxsackievirus and adenovirus receptor have been identified on the cell membrane of the kidney [15], the host receptor allowing these viruses to enter into the cells [25,26]. Moreover, another receptor ubiquitously expressed in renal tissue, namely scavenger receptor class B member 2 (also known as lysosomal integral membrane protein II and CD36b like-2), permit the entry of enteroviruses, including enterovirus 71 and coxsackievirus, into the kidney [16,27].

Incidence of nephrotic syndrome varies by ethnicity, race, and geographic region [1,2,28] and is more common in boys than in girls (male: female ratio range of 1.2:1–2.5:1) [1,28]. An epidemiologic study in Taiwan revealed an average incidence rate of 5.66 per 100,000 children per year [4], higher than most reports from Western countries [28–30]. In addition, a male preponderance (male: female ratio, 1.91:1) of nephrotic syndrome was demonstrated in Taiwan [4]. In our study, the multivariable model revealed that boys had a 1.23-fold increased risk of nephrotic syndrome compared with girls, and the reason for this difference remains unclear.

Association between allergic diseases and nephrotic syndrome has been reported [31–34]. Meadow et al. first described a higher incidence of allergy in children with corticosteroid-responsive nephrotic syndrome [34]. Immune responses are critical in the pathogenesis of both allergic diseases and nephrotic syndrome [35]. In our study, patients with allergic diseases did not have a significantly higher risk of nephrotic syndrome after adjustment for age, sex, urbanization level, parental occupation, and enteroviral infection status. The risk for the development of nephrotic syndrome in enterovirus-infected children was only significant among those without allergic diseases. This inconsistency between our study and earlier studies may be due to differences in the study design. The hygiene hypothesis has suggested a significant inverse association between allergy and exposure to enteroviruses [36,37]. In this study, we identified enteroviral infection to be a significant factor for development of nephrotic syndrome. When being taken into consideration of enterovirus, the link between allergy and nephrotic syndrome was not significant. Additional studies are warranted to elucidate the interaction among enteroviruses, allergy, and nephrotic syndrome.

Despite this study being a nationwide surveillance based on a large sample with comprehensive demographic characteristics, it has several limitations. First, we excluded cases with underlying causes of secondary nephrotic syndrome. However, some risk factors, such as genetic defect and toxic drugs, were unavailable in the database, and these factors can possibly bias the results of this study. Second, the NHIRD lacks information on histopathologic changes of nephrotic syndrome, namely minimal change disease, focal segmental glomerulosclerosis, and membranous nephropathy. Therefore, we could not analyze the association of enteroviral infections with these subtypes of nephrotic syndrome. Third, the included patients of enteroviral infection in this study were based on the ICD-9-CM codes of the NHIRD. The coding and diagnosis of enteroviral infection were made by the clinical physicians. Some of the diagnoses may be based on the symptoms, and others may be based on laboratory confirmation. The database lacked laboratory information about the specimens (stool, blood, cerebrospinal fluid, and nasopharyngeal swab) and the diagnostic techniques (direct fluorescent antibody method, serology, culture, and polymerase chain reaction). Fourth, hand-foot-and-mouth and herpangina, which are the most common types of enteroviral infection, could be diagnosed based on the clinical manifestations. However, a recent study showed that enterovirus could be detected by polymerase chain reaction in only 68.9% of patients with hand-foot-and-mouth and herpangina [38]. The enterovirus prevalence in this study was probably overestimated. On the other hand, many other enteroviral infections are non-specific, such as gastroenteritis, respiratory, and meningitis. The prevalence of enteroviral infection could be underestimated if there was no laboratory confirmation. A recent study using protein microarrays that enables the detection of antiviral antibodies against individual viral proteins from different viral strains may give us much more accurate information of viral infection [39]. Fifth, children with enteroviral infections can be asymptomatic or carriage. Witsø *et al*. [40] reported that human enterovirus was detected in the stool of 51.3% healthy infants. Moreover, enterovirus can cause respiratory illnesses in children, including nonspecific upper respiratory tract infections, bronchitis, bronchiolitis, and pneumonia [41]. It is likely that a proportion of children with nephrotic syndrome in the non-enterovirus-infected cohort had pauci- or asymptomatic enteroviral infection. This could influence the results of our study. Finally, this study used a database relying on ICD-9-CM diagnostic codes, and inaccurate coding maybe present although several studies have proved the accuracy of the database [42,43].

## Conclusions

Nephrotic syndrome is a critical renal disease in children. This large nationwide cohort study revealed an association between enteroviral infection and nephrotic syndrome. Additional studies are warranted to understand the pathogenesis of enteroviral infections on nephrotic syndrome.

## Abbreviations

HIV: human immunodeficiency virus
NHIRD: National Health Insurance Research Database
ICD-9-CM: International Classification of Diseases, Ninth Revision, Clinical Modification
HR: hazard ratio
CI: confidence interval
